# *Bacillus xiamenensis* Inhibits the Growth of *Moraxella osloensis* by Producing Indole‐3‐Carboxaldehyde

**DOI:** 10.1002/mbo3.70009

**Published:** 2024-11-13

**Authors:** Masahiro Watanabe, Yuika Sekino, Kouji Kuramochi, Yuuki Furuyama

**Affiliations:** ^1^ Department of Applied Biological Science, Faculty of Science and Technology Tokyo University of Science Noda‐shi Chiba Japan

**Keywords:** *Bacillus xiamenensis*, growth inhibition, indole‐3‐carboxaldehyde, *Moraxella osloensis*, skin health

## Abstract

*Moraxella osloensis*, a gram‐negative rod‐shaped bacterium found on human skin, produces 4‐methyl‐3‐hexenoic acid, contributing to clothing and body malodor. *M. osloensis* is resistant to UV light, drying, and antimicrobials, making its eradication challenging. As the skin is low in nutrients, commensal bacteria compete for resources and use diverse strategies to inhibit their competitors. Therefore, skin‐derived bacteria that exhibited growth‐inhibitory activity against *M. osloensis* were searched. Screening skin‐derived bacteria using a coculture halo assay revealed that *Bacillus xiamenensis* formed an inhibition zone with *M. osloensis*. Coculture plates were extracted with ethyl acetate and fractionated using a silica gel column and preparative thin‐layer chromatography to isolate the active compound from the *B. xiamenensis* metabolites. Nuclear magnetic resonance spectroscopy identified the active compound as indole‐3‐carboxaldehyde, which has low toxicity in humans. At soluble concentrations, indole‐3‐carboxaldehyde does not inhibit the growth of other bacteria, such as *Staphylococcus aureus*, *Escherichia coli*, and *Bacillus subtilis*, suggesting *M. osloensis* is highly sensitive to indole‐3‐carboxaldehyde. These findings highlight *B. xiamenensis* as a promising candidate for the development of a skin probiotic to promote skin health and combat malodor‐causing bacteria.

## Graphical abstract

*Bacillus xiamenensis* interacts with *Moraxella osloensis*, and the indole‐3‐carboxaldehyde produced by *B. xiamenensis* is a promising agent with specific bactericidal activity against *M. osloensis*. structure–activity relationship analysis indicates that the indole ring and aldehyde moiety of indole‐3‐carboxaldehyde are vital for its anti‐*M. osloensis* activity.

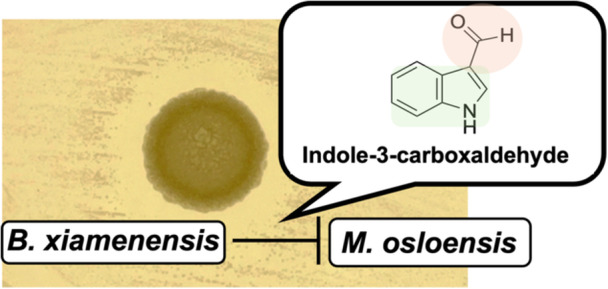

## 1 Introduction

Diverse commensal bacteria inhabit the human skin, forming an ecosystem known as the skin microbiota; its composition varies depending on body location and the properties of the skin. The skin microbiota is balanced on complex interactions among commensal bacteria. Recent research highlights the crucial role of the skin microbiota in maintaining human health, similar to the well‐established influence of the intestinal microbiota (Nakatsuji et al. 2017; Huang, Jiang, and Scott 2022; Timm et al. 2020).

However, some skin commensals sometimes contribute to negative phenomena such as malodors, including sweat odor and the unpleasant “wet‐and‐dirty‐dustcloth‐like” odor associated with laundry (Takeuchi et al. 2012; Kubota et al. 2012). *Moraxella osloensis*, a gram‐negative rod‐shaped bacterium inhabiting human skin, is a causative agent for such malodors. It produces 4‐methyl‐3‐hexenoic acid, the compound responsible for laundry malodor (Kubota et al. 2012). Furthermore, this bacterium is involved in infectious diseases of immunocompromised adults or adults with an underlying condition and healthy children (Lim et al. 2018). Eradicating this bacterium is challenging due to its resistance to UV light, drying, and even antimicrobial laundry detergents containing sodium hypochlorite or quaternary ammonium salts (Kubota et al. 2012; Bouassida et al. 2018). Their antimicrobial activity can be enhanced by a high concentration of surfactants. However, clothing degradation, toxicity to the body, and environmental impact are some factors preventing its applications (Bouassida et al. 2018).

In this study, we focused on the microbial interaction system in the skin microbiota and explored the potential of commensal skin bacteria to establish a new control strategy for the malodor‐causing agent *M. osloensis*. As the skin is low in nutrients, commensal bacteria compete for resources and use diverse strategies to inhibit their competitors. For example, *Staphylococcus lugdunensis*, a commensal skin bacterium found mainly in the nasal cavity, has been reported to produce lugdunin to specifically inhibit the growth of *Staphylococcus aureus* (Zipperer et al. 2016). Likewise, other *Staphylococcus* spp. produce lantibiotics for *S. aureus* inhibition (Götz et al. 2014). For another example, *Bacillus* spp., such as *Bacillus subtilis* and *B. coagulans*, affect the skin microbiome and can inhibit skin pathogens such as *Cutibacterium acnes* (Moskovicz et al. 2021; Shan et al. 2021; Majeed et al. 2020).

In this study, we investigated bacteria derived from human skin for their growth‐inhibitory activity against *M. osloensis*. We found that *Bacillus xiamenensis* showed inhibitory activity against *M. osloensis* and revealed the causative inhibitory compound to be indole‐3‐carboxaldehyde. This compound is a known metabolite of intestinal bacteria and has beneficial effects without toxicity in humans (Zelante et al. 2013; Puccetti et al. 2021; Zelante et al. 2021; D'Onofrio et al. 2021). Our findings suggest the potential of *B. xiamenensis* as a skin probiotic that will have a minimal impact on clothing, human health, and the environment.

## 2 Materials and Methods

### 2.1 Bacterial Strains, Media, and Culture Conditions

Skin‐derived bacteria were cultured from a stock collection built in a previous study (Sekino et al. 2024). The *M. osloensis* NBRC 111460 strain was purchased from the Biological Resource Center, National Institute of Technology and Evaluation, Japan. All bacterial strains were cultured on Gifu anaerobic medium (GAM) agar plates or in GAM broth (Shimadzu Diagnostics, Tokyo, Japan) under aerobic conditions at 37°C.

### 2.2 Screening for Skin Commensal Bacteria That Inhibit *M. osloensis*

*M. osloensis* and skin bacteria were precultured overnight in GAM broth at 37°C with shaking (137 rpm) under aerobic conditions. Then, 100 μL of the *M. osloensis* culture was spread onto a GAM agar plate, followed by spotting 10 μL of each skin bacterial culture broth onto the GAM agar plate surface. After 24 h of aerobic incubation at 37°C, the plates were examined for inhibition zones surrounding the spotted skin bacterial colonies.

### 2.3 Identification of the Active Strain via 16S rRNA Gene Sequencing

The bacterial strain exhibiting inhibitory activity (active strain) against *M. osloensis* was identified via 16S rRNA sequencing. Genomic DNA was extracted before the 16S rRNA gene was amplified via PCR using KOD Fx Neo (TOYOBO, Tokyo, Japan) and two universal primers, 27F and 1492R. The resulting PCR products were sequenced using the universal primers 518F and 800R. The 16S rRNA gene sequences were then analyzed using the Basic Local Alignment Search Tool (BLAST) to identify the closest strain. The analysis confirmed the active strain as *B. xiamenensis*.

### 2.4 Extraction of Active Components From Coculture Plates

GAM agar plugs containing the inhibition zone from the coculture plates were excised with a spatula and extracted with ethyl acetate at 4°C overnight. The extracts were filtered, concentrated via evaporation, and stored for further analysis. For controls, sterile GAM plates or plates containing *B. xiamenensis* monocultures were processed similarly, extracting approximately the same amount of medium using a spatula. Additionally, 10 mL of *B. xiamenensis* culture broth was extracted with an equal volume of ethyl acetate.

### 2.5 Evaluation of Growth‐Inhibitory Activity Using the Microdilution Method

The crude ethyl acetate extract or fractions obtained from preparative thin‐layer chromatography (TLC) were dissolved in GAM broth at a final concentration of 2 mg/mL. Aliquots (100 μL) of these fractions were added to a 96‐well plate, and 5 μL of *M. osloensis* culture broth (OD_600_ = 0.2) was inoculated into each well. After incubation for 16 h, the turbidity of each well was measured using a plate reader (Thermo Fisher Scientific, Waltham, MA, USA). Commercially available indole‐3‐carboxaldehyde (Tokyo Kasei Kogyo, Tokyo, Japan) was dissolved in GAM broth at final concentrations ranging from 2 to 1024 mg/mL and assayed as described above. For the selectivity tests, 5 μL of *S. aureus, Escherichia coli*, and *B. subtilis* culture broths (OD_600_ = 0.2) was inoculated into each well. Kanamycin (100 µg/mL) was used as a positive control. To perform structure–activity relationship analysis, commercially available indole‐3‐carboxaldehyde, indole‐3‐carboxylic acid (Tokyo Kasei), indole‐3‐acetic acid (Tokyo Kasei), benzaldehyde (Tokyo Kasei), and indole (Tokyo Kasei) were dissolved in acetone at concentrations of 102.4 mg/mL, and 1% acetone solution was added to *M. osloensis* culture broth at a final concentration of 1024 μg/mL.

### 2.6 Purification and Structural Identification of Indole‐3‐Carboxaldehyde

The ethyl acetate extracts of coculture plates were fractionated using silica gel column chromatography with a gradient of EtOAc/MeOH (1:0, 4:1, 1:1, and 1:4). The active fraction was further purified using preparative TLC (PLC Silica gel 60 F_254_ plates; 0.5 mm) using CHCl_3_/MeOH (15:1). Compounds were visualized under UV light (254 nm). Structural identification of the active compound as indole‐3‐carboxaldehyde was confirmed via nuclear magnetic resonance (NMR) spectroscopy. NMR spectra were recorded on a Bruker Avance 400 spectrometer at 400 MHz for ^1^H NMR using chloroform‐*d* as the solvent.

Chemical shifts for indole‐3‐carboxaldehyde: ^1^H NMR (400 MHz, CDCl_3_) with *δ* 10.08 (s, 1H), 8.80 (br s, 1H), 8.34–8.32 (m, 1H), 7.86 (d, *J* = 3.3 Hz, 1H), and 7.46–7.44 (m, 1H), 7.35–7.33 (m, 2H); ^13^C NMR (100 MHz, CDCl₃) with *δ* 185.2, 136.6, 135.2, 124.5, 124.4, 123.0, 122.0, 119.8, and 111.5.

### 2.7 Statistical Analysis

All experimental results are expressed as the means ± standard deviation of three independent experiments. All data were analyzed using MS Office Excel (version 2406) (Microsoft Corp., Redmond, WA, USA).

## 3 Results

### 3.1 *B. xiamenensis* Inhibits the Growth of *M. osloensis*

**Figure 1:**
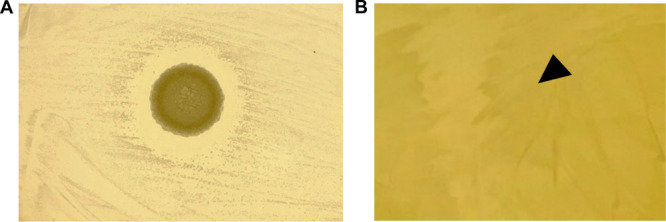
*Bacillus xiamenensis* formed an inhibition zone against *Moraxella osloensis*. (A) The culture supernatant from *B. xiamenensis* spotted onto a precultured Gifu anaerobic medium (GAM) plate containing *M. osloensis* formed an inhibition zone after overnight culture. (B) The sterile GAM media control did not inhibit *M. osloensis* growth; the black triangle indicates the media dropping point.

The inhibitory potential of human‐skin‐derived bacteria against *M. osloensis* was investigated in the present study. In a previous study, a stock collection of bacteria sampled from human skin containing commensal and temporally attached bacteria was established (Sekino et al. 2024). Using these stocks, bacteria with growth‐inhibitory activity against *M. osloensis* were screened using a coculture assay. Each bacterium was cultured overnight in liquid GAM, and the supernatant was dropped onto a GAM plate containing *M. osloensis*. Following overnight coculture, an inhibition zone was formed, seen as a bacteria‐induced halo against *M. osloensis* (Figure 1). Phylogenetic analysis using 16S rRNA gene sequencing identified this bacterium as *B. xiamenensis*.

### 3.2 Isolation of the Active Compound Produced by *B. xiamenensis*

**Figure 2:**
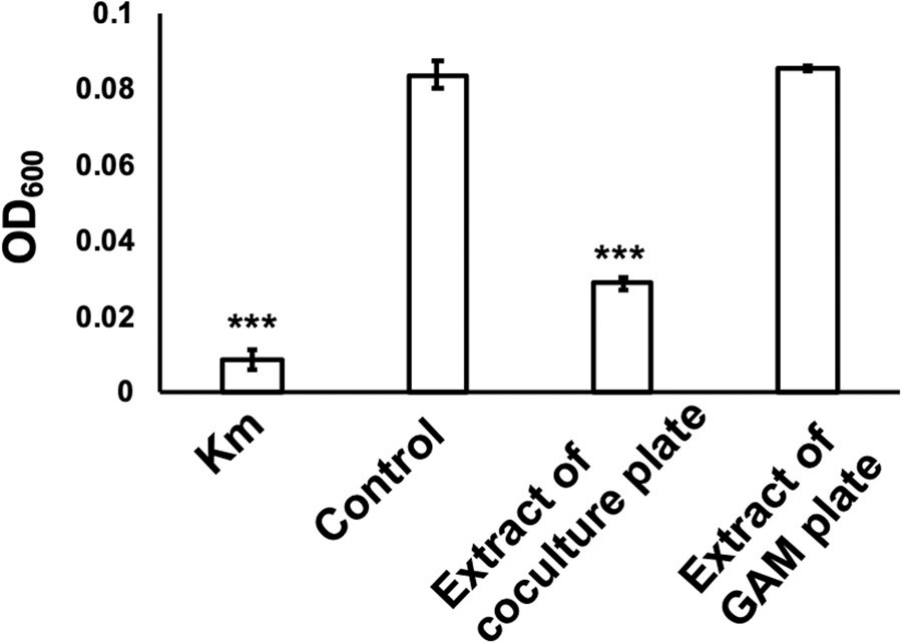
Inhibitory activity of coculture extracts against *Moraxella osloensis*. The ethyl acetate extract from coculture plates containing *Bacillus xiamenensis* and *M. osloensis* exhibited inhibitory activity against *M. osloensis*, as evidenced by measuring the optical density at 600 nm (OD_600_) in a microdilution assay. Sterile GAM broth (control) and the extract from the sterile GAM plate (extract of GAM plate) did not inhibit *M. osloensis*. Kanamycin (100 µg/mL) was used as the positive control (Km). The data represent experiments conducted in triplicate. Error bars represent the standard deviation (SD). ***p < 0.001.

**Figure 3:**
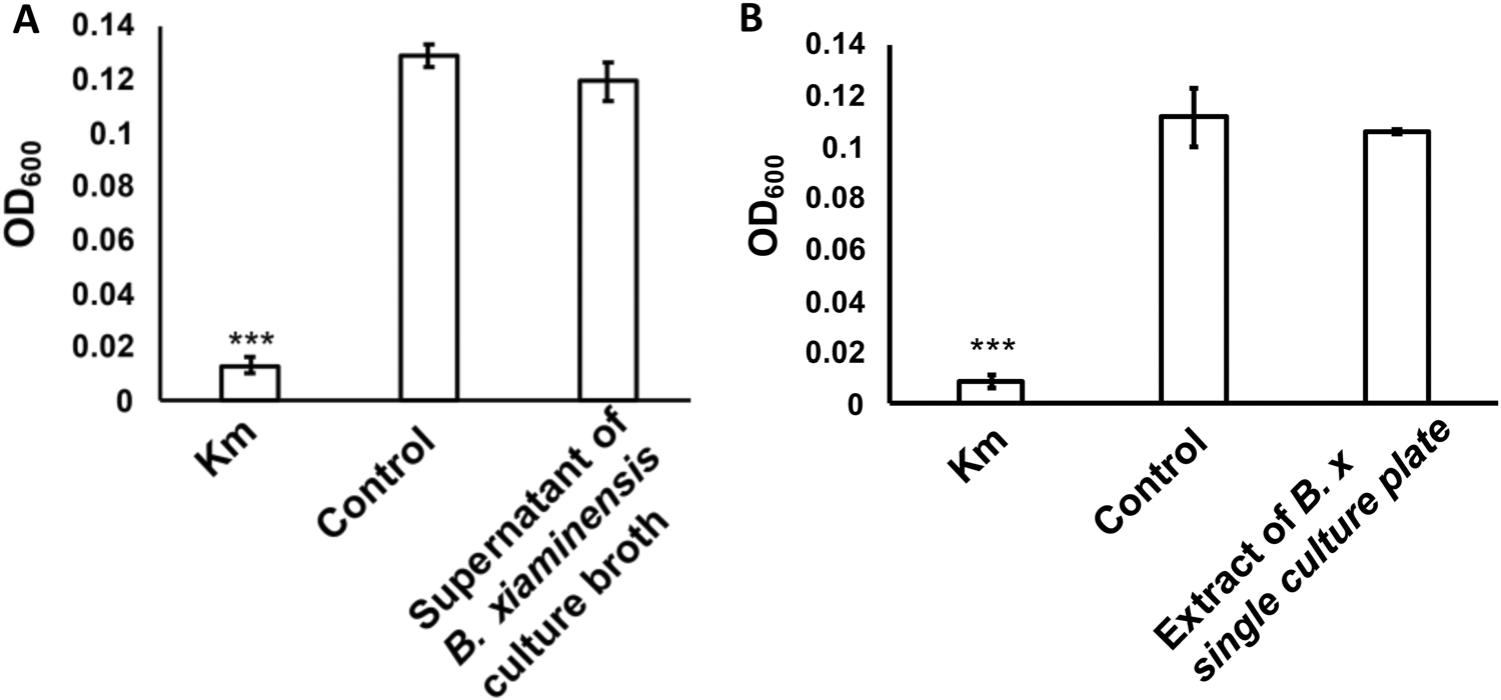
Inhibitory activity of the ethyl acetate extract of *Bacillus xiamenensis* monocultures. The ethyl acetate extract from *B. xiamenensis* monocultures, either from the culture broth (A) or plates (B), did not inhibit the growth of *Moraxella osloensis*, as determined by their OD_600_. Kanamycin (100 µg/mL) was used as the positive control (Km), and sterile GAM broth was used as the negative control (Control). The data represent experiments conducted in triplicate. Error bars represent the SD. ***p < 0.001.

**Figure 4:**
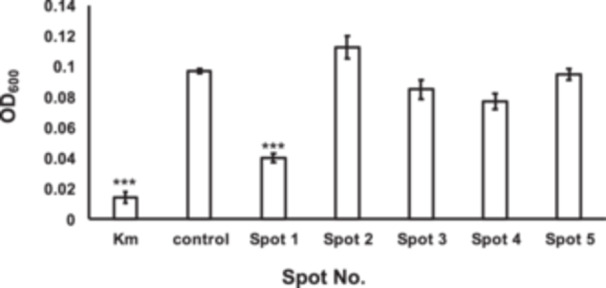
Isolation of the active (inhibitory) fraction from coculture plates by preparative thin‐layer chromatography (TLC). Fractions obtained from the preparative TLC of the coculture plates extract were assayed for inhibitory activity against *Moraxella osloensis* using a microdilution assay. Fraction No. 1 (Spot 1) showed the strongest inhibitory effect against *M. osloensis*, as measured from the OD_600_. Kanamycin (100 µg/mL) was used as the positive control (Km), and sterile GAM broth was used as the negative control (Control). The data represent experiments conducted in triplicate. Error bars represent the SD. ***p < 0.001.

Coculture plates were extracted with ethyl acetate to isolate the active compounds. The activity of this extract was confirmed using the microdilution method (Figure 2). The liquid (Figure 3A) and agar medium (Figure 3B) extracts, in which *B. xiamenensis* was cultured alone (monoculture), lacked inhibitory activity. Further bioactivity‐guided fractionation of the ethyl acetate extract of coculture plates was conducted using silica gel column chromatography, and preparative TLC yielded the active fraction (Figure 4).

### 3.3 Identification of the Active Compound as Indole‐3‐Carboxaldehyde

**Figure 5:**
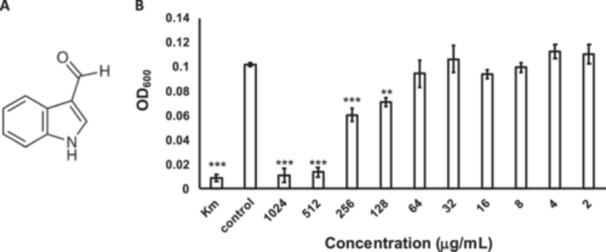
Indole‐3‐carboxaldehyde was identified as the active compound. (A) Chemical structure of indole‐3‐carboxaldehyde. (B) The inhibitory activity of indole‐3‐carboxaldehyde was determined by measuring the OD_600_ in a microdilution assay. Kanamycin (100 µg/mL) was used as the positive control (Km), and sterile GAM broth was used as the negative control (Control). The data represent experiments conducted in triplicate. Error bars represent the SD. **p < 0.01; ***p < 0.001.

**Figure A1:**
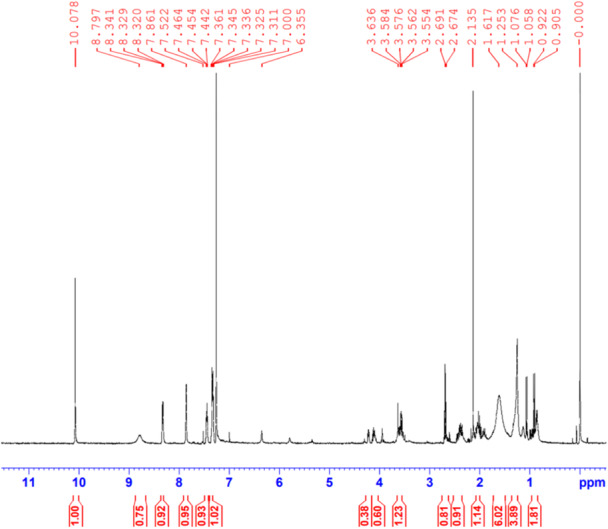
^1^H NMR (400 MHz, CDCl₃) spectra of indole‐3‐carboxaldehyde purified from culture broth.

**Figure A2:**
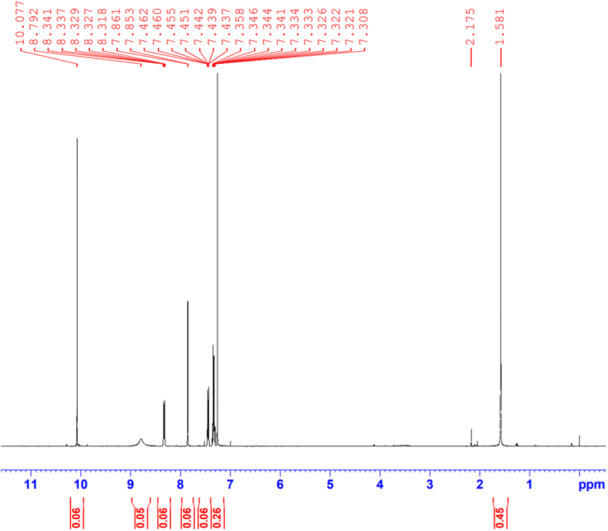
^1^H NMR (400 MHz, CDCl₃) spectra of commercially available indole‐3‐carboxaldehyde.

**Figure A3:**
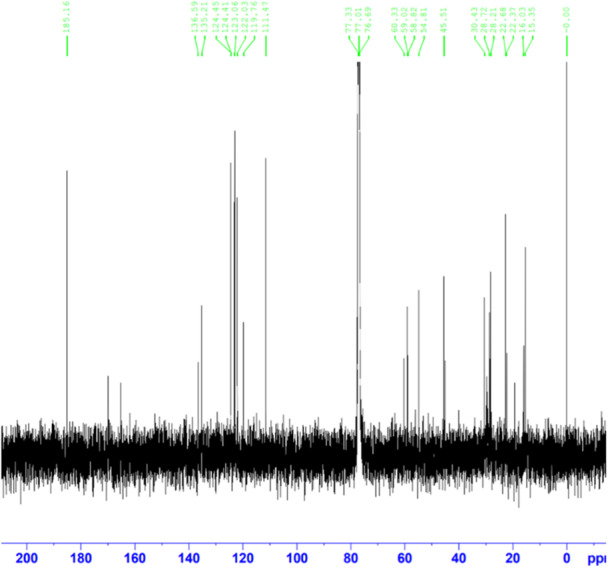
^13^C NMR (100 MHz, CDCl₃) spectra of isolated indole‐3‐carboxaldehyde.

**Figure A4:**
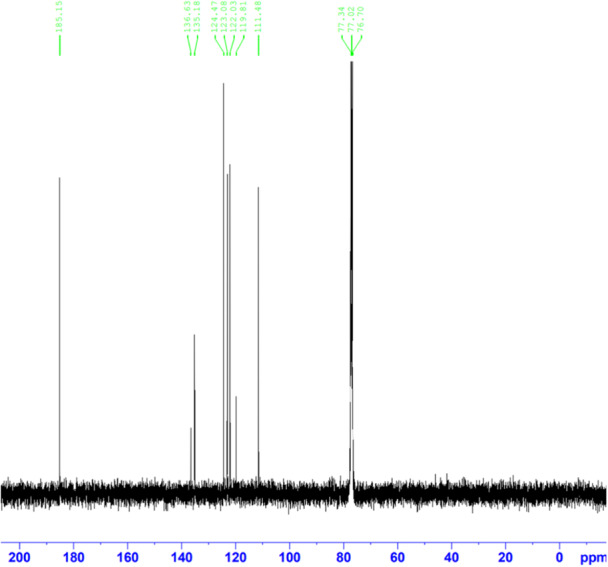
^13^C NMR (100 MHz, CDCl₃) spectra of purchased indole‐3‐carboxaldehyde.

Structural analysis using ^1^H NMR spectroscopy identified the compound in the active fraction as indole‐3‐carboxaldehyde (Figures 5A, A1, A2, A3, A4) (Puccetti et al. 2021; Carrasco et al. 2020). To the best of our knowledge, this is the first report showing that *B. xiamenensis* produces indole‐3‐carboxaldehyde. We also evaluated the effect of commercially available indole‐3‐carboxaldehyde on *M. osloensis* and found that it inhibits the growth of *M. osloensis* in a similar manner (Figure 5B). At concentrations of 521–1024 μg/mL, indole‐3‐carboxaldehyde exhibited relatively high inhibitory activity to *M. osloensis*.

### 3.4 Indole‐3‐Carboxaldehyde Does Not Inhibit the Growth of *S. aureus*, *E. coli*, and *B. subtilis* at Soluble Concentrations

**Figure 6:**
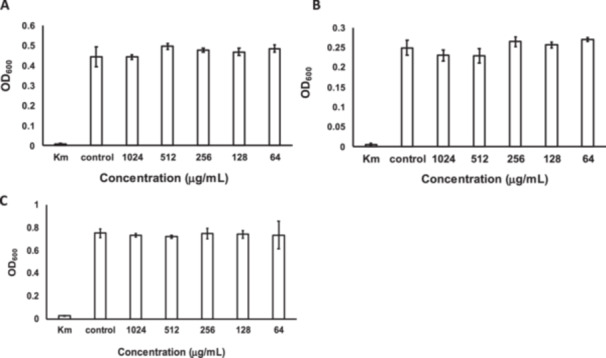
Selective inhibition of *Moraxella osloensis* by indole‐3‐carboxaldehyde. Indole‐3‐carboxaldehyde did not inhibit the growth of (A) *Staphylococcus aureus*, (B) *Escherichia coli*, or (C) *Bacillus subtilis*, as determined by measuring the OD_600_ in a microdilution assay. Kanamycin (100 µg/mL) was used as the positive control (Km), and sterile GAM broth was used as the negative control (Control). The data represent experiments conducted in triplicate. Error bars represent the SD.

**Figure A5:**
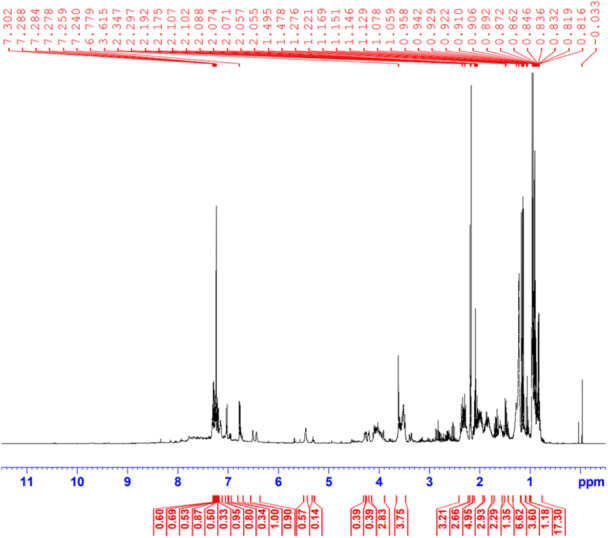
^1^H NMR (400 MHz, CDCl₃) spectra of extract from coculture plate of *Moraxella osloensis* and *Bacillus subtilis*.

**Figure A6:**
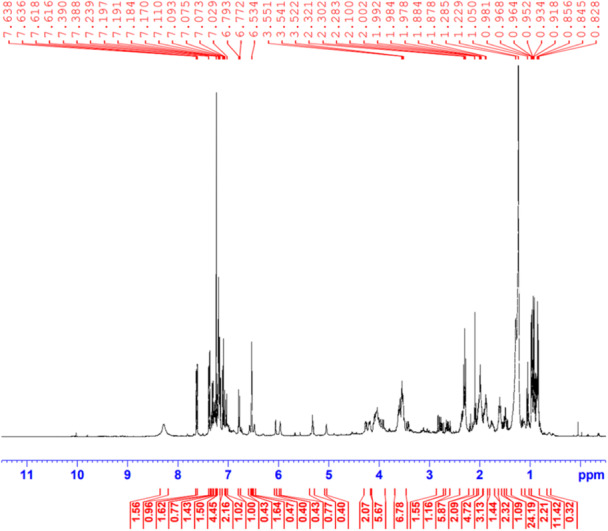
^1^H NMR (400 MHz, CDCl₃) spectra of extract from coculture plate of *Moraxella osloensis* and *Escherichia coli*.

**Figure A7:**
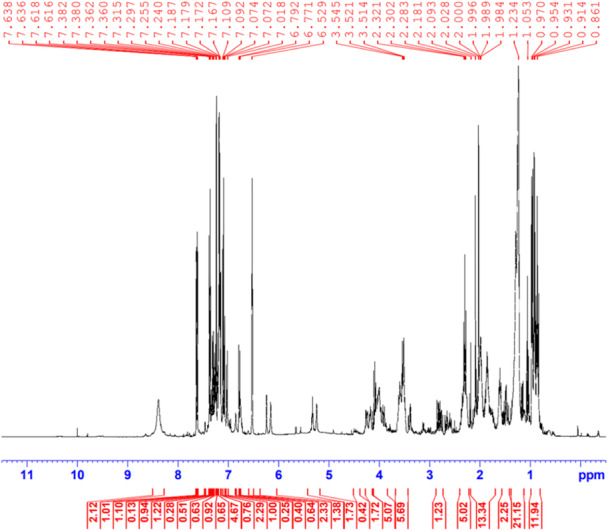
^1^H NMR (400 MHz, CDCl₃) spectra of extract from coculture plate of *Moraxella osloensis* and *Staphylococcus aureus*.

**Figure A8:**
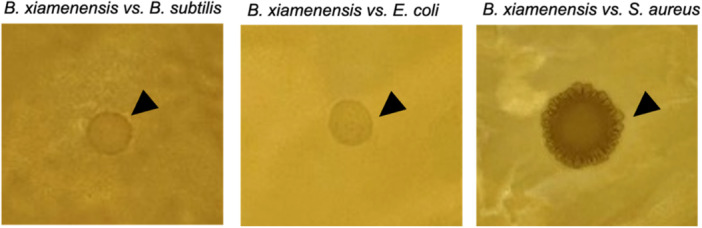
Coculture plates of other bacteria.

**Figure A9:**
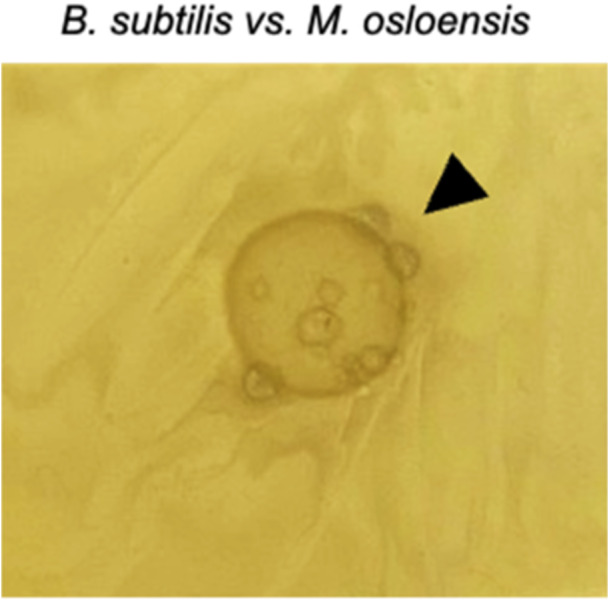
Coculture plates of *Bacillus subtilis* and *Moraxella osloensis*.

To examine the spectrum of indole‐3‐carboxaldehyde activity, microdilution assays were performed on *S. aureus*, *E. coli*, and *B. subtilis*. Indole‐3‐carboxaldehyde did not inhibit the growth of these three bacteria at 1024 μg/mL (Figure 6). Solubility limitations in water (2048 μg/mL and above) prevented testing higher concentrations. This result indicates that *M*. *osloensis* is more sensitive to indole‐3‐carboxaldehyde at concentrations below 1024 μg/mL than the other bacteria used in this study. When *B. xiamenensis* was cocultured with *S. aureus*, *E. coli*, and *B. subtilis*, an inhibition zone was not formed (Figure A8). Furthermore, indole‐3‐carboxaldehyde was not detected from the coculture plates of *S. aureus*, *E. coli*, or *B. subtilis* with *B. xiamenensis* (Figures A5, A6, A7). The results indicated that the production of indole‐3‐carboxaldehyde in *B. xiamenensis* was induced by *M. osloensis*. Since Muro et al. reported that the *Bacillus* genus interacted with *M. osloensis*, *B. subtilis* was cocultured with *M. osloensis*. Consequently, *B. subtilis* did not form an inhibitory zone against *M. osloensis* (Figure A9). *B. xiamenensis* was expected to sense *M. osloensis* using a specific signaling pathway.

### 3.5 The Indole Ring and Aldehyde Moiety of Indole‐3‐Carboxaldehyde Are Important to Inhibitory Activity

**Figure 7:**
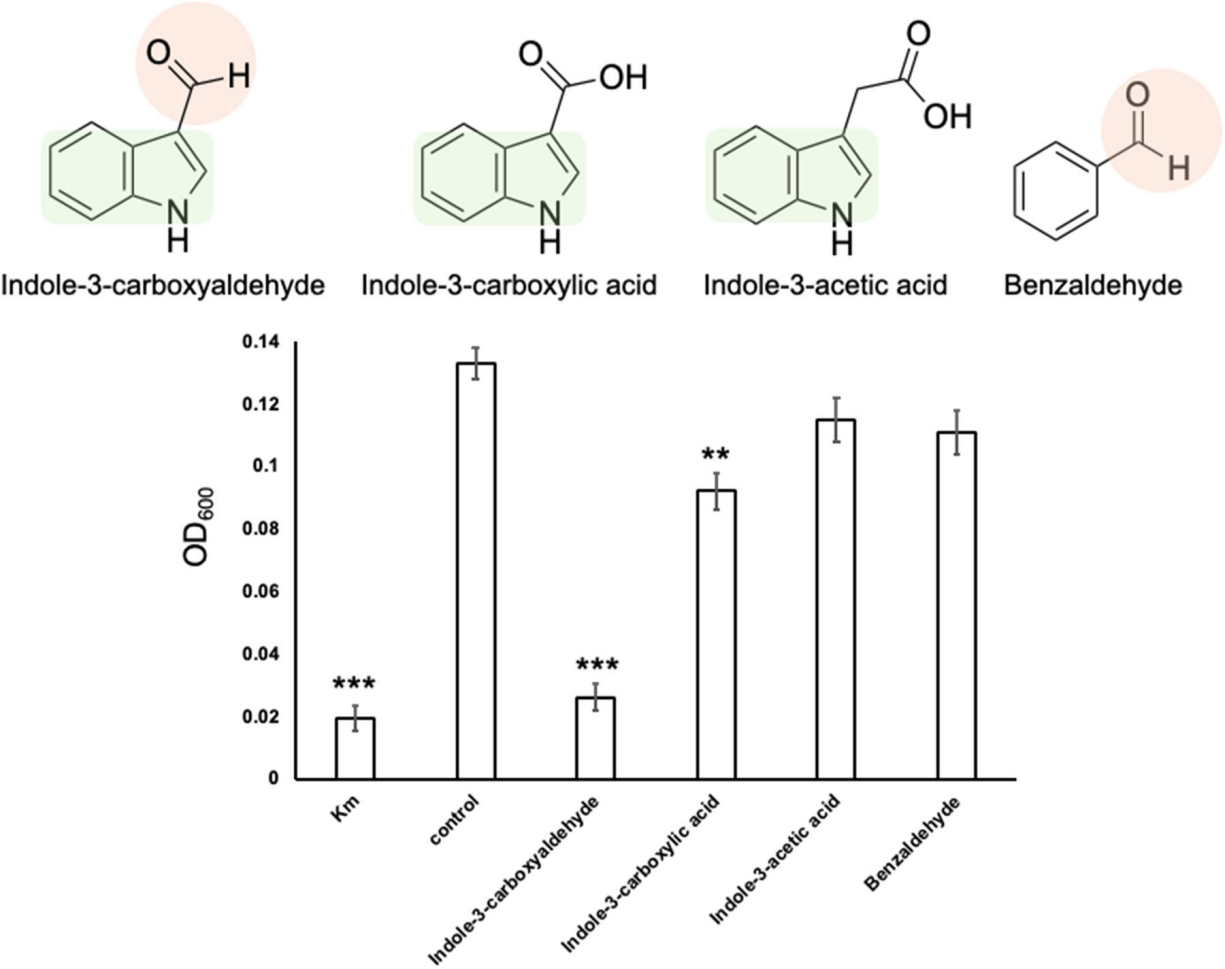
The indole ring and aldehyde moiety of indole‐3‐carboxaldehyde are critical for inhibitory activity. Indole‐3‐carboxylic acid, indole‐3‐acetic acid, and benzaldehyde do not inhibit the growth of *Moraxella osloensis* as determined by measuring the OD_600_ in a microdilution assay. Kanamycin (100 µg/mL) was used as the positive control (Km), and sterile GAM broth was used as the negative control (Control). Each compound was applied with a final concentration of 1024 μg/mL. The data represent experiments conducted in triplicate. Error bars represent the SD. **p < 0.01; ***p < 0.001. The green circle indicates the indole ring and the red circle indicates aldehyde moiety.

**Figure A10:**
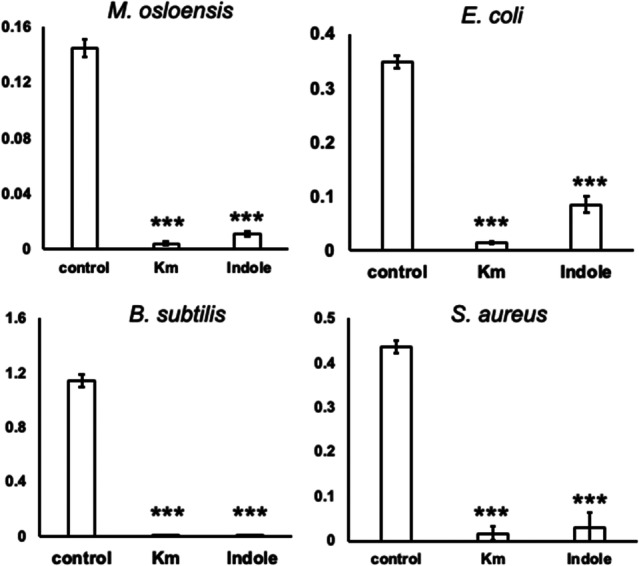
Inhibitory assay conducted using indole. Error bars represent the SD. ***p < 0.001.

Inhibitory assays using indole‐3‐carboxyaldehyde, indole‐3‐carboxylic acid, indole‐3‐acetic acid, and benzaldehyde were used to conduct structure–activity relationship analysis. Indole‐3‐carboxylic acid, indole‐3‐acetic acid, and benzaldehyde showed weak or no inhibitory activity against *M. osloensis* (Figure 7). Conversely, indole inhibited all four bacteria used in the present study (Figure A10). The results indicated that the indole ring and aldehyde moiety of indole‐3‐carboxyaldehyde are vital for the anti‐*M. osloensis* activity.

## 4 Discussion

Indole‐3‐carboxaldehyde is produced predominantly by *Lactobacillus* spp. in the intestine (Zelante et al. 2013). To the best of our knowledge, the antimicrobial activity of indole‐3‐carboxaldehyde has not been reported, although some derivatives have been reported to exhibit such properties (Carrasco et al. 2020; Gurkok, Altanlar, and Suzen 2009). In plants, indole‐3‐carboxaldehyde is converted from indole‐3‐acetonitrile (IAN) by CYP71B6 (Böttcher et al. 2014). Conversely, the bacterial biosynthetic genes for indole‐3‐carboxaldehyde and IAN are largely unknown (Tang et al. 2023). The *Oxd*s genes are bacterial genes associated with IAN biosynthesis (Tang et al. 2023; Sun et al. 2022; Li et al. 2018). Therefore, we searched for *Oxd* homolog, although no homolog was found from the genome of *B. xiamenensis*, which suggests that *B. xiamenensis* biosynthesizes indole‐3‐carboxaldehyde via an unknown pathway. Further analyses, such as genome‐wide gene expression analysis of *B. xiamenensis* under coculture conditions, are required to reveal the indole‐3‐carboxaldehyde biosynthetic pathway in this bacterium.

In the present study, the indole‐3‐carboxaldehyde produced by *B. xiamenensis* showed antibacterial activity against *M. osloensis* at a low concentration range (512–1024 μg/mL). We also attempted to evaluate the activity of indole‐3‐carboxaldehyde at higher concentrations; however, we found that it was not soluble in liquid media at concentrations higher than 1024 μg/mL. From this result, the concentration of indole‐3‐carboxaldehyde produced by *B. xiamenensis* under natural conditions, such as on human skin or in soil, is expected to be below 1024 μg/mL. Since indole‐3‐carboxaldehyde and *B. xiamenensis* did not inhibit the growth of other bacteria, it may act via a specific pathway to inhibit *M. osloensis*. Furthermore, indole‐3‐carboxylic acid, indole‐3‐acetic acid, and benzaldehyde exhibited no or weak inhibitory activity against *M. osloensis*. This indicated that the indole ring and the aldehyde moiety of indole‐3‐carboxyaldehyde are vital for the activity. Since indole exhibits strong inhibitory activity against all four bacteria, the aldehyde moiety is specifically involved in selective activity. The cell membrane of *M. osloensis* has high lipid and fatty acid compositions (Sugimoto et al. 1983; Kubota et al. 2012). The higher hydrophobicity of indole‐3‐carboxaldehyde might be related to the sensitivity of *M. osloensis*. Further studies are required to investigate the precise mechanism of action.

The inhibitory activity against *M. osloensis* was only observed in extracts of coculture plates, not in extracts from *B. xiamenensis* monoculture plates or broths. This suggests that indole‐3‐carboxaldehyde production by *B. xiamenensis* is induced by its interaction with *M. osloensis*. Muro et al. reported that *LuxS* is involved in the interaction between *M. osloensis* and *Bacillus* genera. *LuxS* is a quorum sensing gene in the genus *Bacillus*, including *B. subtilis* (Duanis‐Assaf et al. 2016); however, *B. subtilis* did not inhibit *M. osloensis*, which suggests that *B. xiamenensis* has a specific sensing pathway for *M. osloensis*.

*M. osloensis* has been isolated from human skin (Cosseau et al. 2016), respiratory tracts (Koleri et al. 2022), and mucous membranes (Koleri et al. 2022). It has also been isolated from indoor environments, such as hospitals (Batinovic et al. 2020), restrooms (Meschke et al. 2009), and air conditioners (Batinovic et al. 2020), as well as outdoor locations, such as lakes (Foti et al. 2008), rivers (Escalante et al. 2009), and composting areas (Vaz‐Moreira et al. 2008). *B. xiamenensis* was first isolated from the intestinal tract of fish and is distributed widely in soil and water (Lai, Liu, and Shao 2014; Wagh, Osborne, and Sivarajan 2022). It has been used to remove heavy metals from the environment and to control plant pathogens (Al Farraj and Elshikh 2023). Although *B. xiamenensis* can attach to our skin temporally, the bacterium was not thought to be a resident of human skin. Our findings suggest the possibility of an interaction relationship between the two bacteria. Based on the fact that *M. osloensis* is detected not only on human skin but also from outdoor sites, *B. xiamenensis* and *M. osloensis* can encounter each other in natural environments such as soil and water. Further studies are warranted to confirm the interaction condition of *B. xiamenensis* and *M. osloensis*.

## 5 Conclusions

*M. osloensis* is a bacterium that causes unpleasant clothing odors and is difficult to eradicate with conventional methods such as detergents and UV light. In this study, we identified *B. xiamenensis* interacting with *M. osloensis*, and the indole‐3‐carboxaldehyde produced by *B. xiamenensis* as a promising agent with specific bactericidal activity against *M. osloensis*. Indole‐3‐carboxaldehyde has reportedly low toxicity in humans. The results suggest that indole‐3‐carboxaldehyde could be applied to control *M. osloensis*, with minimal environmental impact and low human toxicity. Furthermore, the sensing of *M. osloensis* by *B. xiamenensis* occurs via an unknown gene pathway, which should be explored in future studies.

## Author Contributions

**Masahiro Watanabe:** writing–original draft, methodology. **Yuika Sekino:** methodology. **Kouji Kuramochi:** writing–review and editing, supervision. **Yuuki Furuyama:** writing–review and editing, conceptualization, project administration.

## Ethics Statement

The authors have nothing to report.

## Conflicts of Interest

The authors declare no conflicts of interest.

## Data Availability

The data that support the findings of this study are openly available in Zenodo: https://doi.org/10.5281/zenodo.13989680. Sequence data are openly available in the NCBI GenBank under accession PQ516773: https://www.ncbi.nlm.nih.gov/nuccore/PQ516773.
